# Development of a consensus operational definition of child assent for research

**DOI:** 10.1186/s12910-017-0199-4

**Published:** 2017-06-09

**Authors:** Alan R. Tait, Michael E. Geisser

**Affiliations:** 1 Departments of Anesthesiology, University of Michigan Health System, 1500 E. Medical Center Drive, Ann Arbor, MI 48109 USA; 2 Physical Medicine and Rehabilitation, University of Michigan Health System, 1500 E. Medical Center Dr, Ann Arbor, USA; 3 Center for Bioethics and Social Sciences in Medicine, University of Michigan Health System, 1500 E. Medical Center Dr, Ann Arbor, USA; 4 Michigan Institute for Clinical & Health Research, University of Michigan Health System, 1500 E. Medical Center Dr, Ann Arbor, USA

**Keywords:** Assent, Definition, Children, Adolescents

## Abstract

**Background:**

There is currently no consensus from the relevant stakeholders regarding the operational and construct definitions of child assent for research. As such, the requirements for assent are often construed in different ways, institutionally disparate, and often conflated with those of parental consent. Development of a standardized operational definition of assent would thus be important to ensure that investigators, institutional review boards, and policy makers consider the assent process in the same way. To this end, we describe a Delphi study that provided consensus from a panel of expert stakeholders regarding the definitions of child assent for research.

**Methods:**

Based on current guidelines, a preliminary definition of assent was generated and sent out for review to a Delphi panel including pediatric bioethicists and researchers, Institutional Review Board members, parents, and individuals with regulatory/legal expertise. For each subsequent review, the process of summarizing and revising responses was repeated until consensus was achieved. Panelists were also required to rank order elements of assent that they believed were most important in defining the underlying constructs of the assent process (e.g., capacity for assent, disclosure). In providing these rankings, panelists were asked to frame their responses in the contexts of younger (≤ 11 yrs) and adolescents/older children (12-17 yrs) in non-therapeutic and therapeutic trials. Summary rankings of the most important identified elements were then used to generate written construct definitions which were sent out for iterative reviews by the expert panel.

**Results:**

Consensus regarding the operational definition was reached by 14/18 (78%) of the panel members. Seventeen (94%) panelists agreed with the definitions of capacity for assent, elements of disclosure for younger children, and the requirements for meaningful assent, respectively. Fifteen (83%) members agreed with the elements of disclosure for adolescents/older children.

**Conclusions:**

It is hoped that this study will positively inform and effect change in the way investigators, regulators, and IRBs operationalize the assent process, respect children’s developing autonomy, and in concert with parental permission, ensure the protection of children who participate in research.

**Electronic supplementary material:**

The online version of this article (doi:10.1186/s12910-017-0199-4) contains supplementary material, which is available to authorized users.

## Background

While research participation for most children requires the permission of parent(s)/guardian(s), the bioethical principle of “respect for persons” requires that children also provide their assent to participate to the degree that they are deemed “capable of providing it,” taking into account the child’s “age, maturity, and psychological state” (45CFR46.608a) [1]. While this regulatory framework is designed to allow children to express their burgeoning autonomy, foster moral growth, and develop decision-making skills, it provides absolutely **no** practical guidance with respect to *what* actually constitutes assent and *which* children are capable of providing it [2, 3]. Indeed, Unguru et al. wrote: “Despite significant progress there is still considerable disagreement surrounding many fundamental components of assent. No consensus exists in guidelines, as disseminated by the federal government, professional associations, or by the assent community [4].” Because of this lack of consensus, the requirements for assent (e.g., how capacity for assent should be assessed, what information children actually want, and what should be disclosed) are often construed in different ways, institutionally disparate, and often inappropriately conflated with those of parental consent [5, 6].

Federal guidelines (45CFR46.102a) [1] define *Assent* as “a child’s affirmative agreement to participate in research” which does not take into consideration the complexity of the assent process. Furthermore, because there is no consensus regarding the definition of assent, individual IRBs often apply different standards for assent [7]. For example, arbitrary age-thresholds are often used as proxy measures of children’s ability to assent. While one could argue that an age-based approach protects children from their own lack of experience, it has the potential to ignore the child’s cognitive and psychosocial features [8]. Indeed, children’s understanding of research information can be highly variable across and even within age groups [9]. Recent work however suggests that age explains most of the variance in children’s capacity to consent and that some children over the age of 12 years may be capable of providing their own consent [10, 11]. However, because not all children are little adults, one cannot hold them to the same standards as those of their parents and, as such the requirements for assent must be viewed independently from consent.

In an attempt to provide greater standardization of the assent process, this study was designed to seek consensus from relevant stakeholders with respect to developing a *single*, concise operational definition of child assent for research. We were also interested in obtaining consensus definitions of the underlying constructs of assent as applied to younger and adolescent/older children in both therapeutic and non-therapeutic trials.

## Methods

Delphi has been described as “… a structured process for collecting and distilling knowledge from a group of experts by means of a series of questionnaires interspersed with controlled opinion feedback [12],” and has been used extensively in public health as means of obtaining expert consensus [13]. This study was deemed exempt by the University of Michigan’s Institutional Review Board (IRB). Conducted from September 2015 to May 2016, the study consisted of 3 phases:

### **Phase 1**: Selecting the expert panel

Since the goal of a Delphi panel is to elicit a variety of informed viewpoints we sought expert input from pediatric researchers, IRB members, bioethicists, parents, and individuals with pediatric regulatory/legal expertise. Expertise was determined by peer-reviewed publications, memberships in professional societies, IRBs, regulatory bodies, and parent/child advocacy groups [12]. We included both sexes, minorities (if known), and members from geographically different regions.

Consent to participate was obtained via e-mail. Participants were informed of the voluntary nature of the study and assured that their names would not be revealed to any of the other panel members until publication of the study. Final publication and sharing of the identities of the panel members required written permission.

### **Phase 2**: Generation of a preliminary operational definition of assent

We first performed a literature review of current national and international definitions of assent including, among others, the Code of Federal Regulations [1] the American Academy of Pediatrics [14] and the Royal College of Paediatrics and Child Health [15]. Literature reviews were based on searches using MEDLINE/PubMed and Google Scholar. Based on these reviews, we generated a preliminary operational definition that was presented for consideration to the expert panel.

### **Phase 3**: Round 1 reviews

Each potential Delphi member was sent e-mail instructions regarding expectations and roles. The e-mail also contained the preliminary operational definition together with a brief questionnaire containing four open-ended statements based on the Delphi study of consent comprehension by Buccini et al. [16]:

- “The elements of the proposed definition that I **agree** with include …”
- “The elements of the proposed definition that I did **not agree** with include …”
- “I feel the following elements are essential to retain or add to the proposed definition …”
- “My suggestions for modifying or changing the definition are as follows …”

Each panelist’s responses were e-mailed back to the principal investigator, de-identified on receipt and analyzed anonymously, thereafter. Responses were scrutinized by both authors to identify common themes using standard qualitative techniques i.e., identifying repetitions, similarities and differences. Up to 2 e-mail reminders were sent in the event that a review was not completed within the specified time frame.

### Iterative reviews

*a) Operational Definition:* For each round, the process outlined above of collecting, summarizing, analyzing, and revising panelists’ responses was repeated. At the point wherein the definitions appeared to coalesce, a call for consensus (>75% agreement) was initiated.

*b) Constructs definitions:* We were also interested in obtaining consensus on the underlying constructs of assent e.g., assessment of capacity, disclosure, etc. Because such constructs likely differ depending on the developmental level of the child and the type of study e.g., non-therapeutic vs therapeutic, we asked panelists to consider each definition within these contexts.

Given that it would be impossible to establish individual definitions for all age and developmental levels, for the purposes of this study, we asked panelists to consider children above and below 11 years. While we do not necessarily agree with age-cut-offs per se, our rationale for this “developmental” cut-off was that it corresponds well with established beliefs regarding when children transition from concrete to operational thought [17]. Adolescents begin to understand and develop the ability to act from moral motivations at 11-12 years of age [18] and some may also be capable of providing their own consent [10]. Furthermore, studies have shown that children’s understanding of research information improves significantly after this age [9, 19].

We selected 3 constructs including assessment of capacity, information disclosure, and requirements for meaningful assent [20, 21]. For each construct, we generated a list of elements that best describe these constructs. Panelists were asked to review these elements and then rank each in order of importance (0-100, where 100 = highest importance). The total value of the rankings had to equal 100. For example, for the disclosure construct, panelists were required to rank order risks, procedures, benefits, etc. in the contexts of a child vs adolescent/older child in both a therapeutic and non-therapeutic trial. Summary rankings of the most important elements were then used to generate written definitions which were sent out for iterative reviews by the expert panel.

### Sample size and statistical analysis

Since the basis for selecting the size of a Delphi panel is not a statistical one, conventional power analyses are not relevant [12]. The literature, however, suggests that Delphi projects can be adequately performed with panels of 8-15 experts over 3-4 rounds [22]. Although increasing the sample size can reduce group error somewhat, there is a threshold beyond which increasing the numbers provides only marginal benefit [12]. Furthermore, large panels can become unwieldy and increase the time to consensus. Based on these criteria, our panel of 20 experts would be considered large by most Delphi standards [22]. Open-ended questions were analyzed thematically, as described. Quantitative scoring utilized measures of central tendency e.g., means ± SD, and medians.

## Results

We generated a master list of 33 potential stakeholders of whom 8 failed to respond and 5 responded but declined participation. Of the original pool, 20 agreed to participate. This initial panel comprised of experts in pediatric research (*n* = 11), IRB/regulatory affairs (7), bioethics (9), legal (3), and combinations thereof. Eleven were male and 9 were female and the majority were themselves parents.

### Operational definition

#### Preliminary definition

Based on our synthesis of the current definitions of assent we formulated a preliminary definition of assent retaining some core elements from 45CFR46 [1] as follows:

***“*** *An interactive process between a researcher and child participant involving developmentally appropriate disclosure, discussion, and understanding wherein the child freely affirms their agreement to participate in a proposed research study but has insufficient maturity or lack of legal authority to meet the formal and legal expectations of informed consent. In the absence of an affirmative agreement, mere failure of the child to object should not be construed as assent.”*

#### Round 1 reviews

Nineteen of 20 (95%) panelists responded to this preliminary definition. Terms considered important by the majority included “interactive process,” “developmentally appropriate” and, “affirmative agreement.” A voiced concern was that this definition did not adequately describe what should be disclosed and what the child might expect from the study. Based on themes identified in this first round, a revised definition was generated (see Additional file 1).

#### Round 2 reviews

Thirteen of 20 (68.4%) responded to the first revision. Of concern was the use of the term “free of any outside influence” since parents often exert influence over their child’s decision-making that may not be inherently bad. The revised version from this round is shown in Additional file 1.

#### Round 3 reviews

Seventeen of 20 members (85%) responded to the second revision. A primary concern was the term “must” provide assent. Suggestions were to replace this with “should” and provide caveats under which assent “should” be sought. Others remarked that “what they might expect” was too vague and that children should understand how they might experience what will happen to them e.g., burden/discomfort. The expectations that assent decisions should be “reasonable” and “rational” was eliminated since it was thought to set too high a standard for children. From these comments, a revised version was sent for review (see Additional file 1).

#### Final round reviews

In the final round, 2 panel members were dropped due to loss to follow-up. Eighteen of 18 (100%) members responded to the third revision. At call for consensus, 14/18 (78%) panelists agreed with the operational definition of assent as written. While 2 of the “dissenters” reported that they agreed conceptually with the definition, both suggested minor revisions that were not shared by the majority and thus were not re-negotiated. One member disagreed but offered no justification (we believe that this may have been an error since there were no other indications to suggest disagreement, however, we cannot be certain). Only one member disagreed conceptually believing that the definition held older children to a higher standard. The final version is shown here:

*“Children who lack the legal authority to provide informed consent per state laws should provide their assent to participate in a research study unless they either lack the cognitive ability, their clinical condition precludes their ability to communicate a choice, or the research holds out the prospect of direct benefit that is only available in the context of the research. Assent is an interactive process between a researcher and child participant involving disclosure of cognitively and emotionally appropriate information regarding, at minimum, why the child is being asked to participate, a description of the procedures and how the child might experience them, and an understanding that participation in the study is voluntary. Children should understand that they can decline participation or withdraw from the study at any time. Assent requires that the child explicitly affirms his or her agreement to participate in a manner that reflects their age-appropriate understanding and that is free of undue influence or coercion. In the absence of an explicit agreement, mere failure of the child to object cannot be construed as assent.”*

### Construct definitions

The ranked elements for each of the assent constructs are presented in Table 1. For analysis, we chose a median ranking cut-point of 10 as the demarcation between elements deemed least and most important. While this did not preclude inclusion of elements below this cut-point we used the most important identified elements to generate 4 initial construct definitions of assent i.e., assessment of capacity, disclosure for younger and older children, and requirements for meaningful assent [23]. These initial definitions underwent 2 further revisions by the panel (see Additional file 2).

**Table 1:** Summary of Rankings

Assessment of child capacity	
*Assessment*	*Ranking* *Mean ± SD (Median)*
Discussion with both the parent(s) and child to gauge maturity/cognitive ability	20.33 ± 15.8 (30.0)
Employ “teach back” (feedback) to assess understanding	18.67 ± 20.00 (20.0)
Discussion with the child only to gauge maturity/cognitive ability	12.33 ± 11.3 (10.0)
Based on general developmental level e.g., child versus adolescent	11.67 ± 11.4 (10.0)
Based on age cut-offs: (please specify)	9.00 ± 10.2 (5.0)
Employ a short quiz to determine level of understanding	7.67 ± 10.3 (0.00)
Assessment based on a formal standardized tool e.g., MacCAT-CR	6.00 ± 9.3 (0.0)
Discussion with the parent(s) only to gauge maturity/cognitive ability	3.00 ± 4.6 (0.0)
Disclosure: Non-therapeutic trial-younger children (7-11 yrs)	
*Elements*	*Ranking* *Mean ± SD (Median)*
What will be done (the procedures)	22.56 ± 4.6 (20.0)
That the study is voluntary	16.79 ± 7.2 (20.0)
That they can withdraw at any time	14.49 ± 7.7 (15.0)
Purpose of the study	11.77 ± 7.7 (10.0)
Benefits to the child	6.92 ± 5.9 (10.0)
Risks	6.92 ± 6.9 (5.0)
Benefits to others	5.00 ± 5.0 (5.0)
That the research is different from clinical care	4.00 ± 5.9 (0.0)
Alternatives	3.31 ± 3.7 (3.0)
They do not need to understand, parental permission is sufficient	1.92 ± 3.8 (0.0)
That their information will remain confidential	0.92 ± 1.9 (0.0)
Level of discomfort or effort required	20.0 ± 0.0 (20.0) *n* = 1
Disclosure: Non-Therapeutic trial-older children (12-17 yrs)	
*Elements*	*Ranking* *Mean ± SD (Median)*
What will be done (the procedures)	17.46 ± 6.8 (15.0)
That the study is voluntary	13.07 ± 4.3 (10.0)
Purpose of the study	13.06 ± 5.9 (10.0)
That they can withdraw at any time	11.15 ± 5.1 (10.0)
Risks	10.9 ± 4.9 (10.0)
That the research is different from clinical care	7.69 ± 5.6 (10.0)
Benefits to the child	7.61 ± 3.8 (10.0)
Benefits to others	7.07 ± 4.5 (10.0)
That their information will remain confidential	6.15 ± 5.8 (5.0)
Alternatives	5.00 ± 3.5 (5.0)
They do not need to understand, parental permission is sufficient	0.00 ± 0.00 (0.00)
Level of discomfort or effort required	10.0 ± 0.00 (10.0) *n* = 1
Disclosure: Therapeutic trial-younger children (7-11 yrs)	
*Elements*	*Ranking* *Mean ± SD (Median)*
What will be done (the procedures)	21.25 ± 8.3 (20.0)
Purpose of the study	13.31 ± 8.4 (15.0)
That the study is voluntary	12.41 ± 9.2 (10.0)
That they can withdraw at any time	12.18 ± 10.0 (10.0)
They do not need to understand, parental permission is sufficient	8.08 ± 20.7 (0.0)
Risks	7.46 ± 6.1 (5.0)
Benefits to the child	7.08 ± 5.7 (5.0)
Alternatives	5.00 ± 4.0 (5.0)
That the research is different from clinical care	5.00 ± 6.5 (0.0)
Benefits to others	3.08 ± 3.8 (0.0)
That their information will remain confidential	1.69 ± 3.1 (0.0)
Disclosure: Therapeutic trial-older children (12-17 yrs)	
*Elements*	*Ranking* *Mean ± SD (Median)*
What will be done (the procedures)	17.46 ± 7.6 (17.0)
Purpose of the study	13.84 ± 5.8 (10.0)
That they can withdraw at any time	12.08 ± 5.9 (10.0)
That the study is voluntary	11.38 ± 5.6 (10.0)
Risks	11.2 ± 4.6 (10.0)
That the research is different from clinical care	8.84 ± 5.8 (10.0)
Benefits to the child	7.69 ± 3.9 (10.0)
Alternatives	6.38 ± 3.6 (5.0)
Benefits to others	5.38 ± 4.8 (5.0)
That their information will remain confidential	3.85 ± 3.6 (5.0)
They do not need to understand, parental permission is sufficient	0.00 ± 0.0 (0.0)
Level of discomfort or effort required	10.0 ± 0.00 (10.0) *n* = 1
Requirements for meaningful assent: younger children (7-11 yrs)	
	*Ranking* *Mean ± SD (Median)*
Child should understand the basic information	24.23 ± 10.4 (25.0)
Child should have a developmentally appropriate awareness of their condition	18.84 ± 8.9 (20.0)
Child should be able to appreciate the information (i.e., apply the information to their own situation)	13.84 ± 8.2 (15.0)
Decisions should be free of any external pressure or expectations	13.84 ± 13.6 (10.0)
Child should be able to articulate an unambiguous choice	10.77 ± 6.1(10.0)
Child should be able to reason (i.e., able to incorporate information with personal priorities and potential consequences)	6.54 ± 6.6 (5.0)
Decisions must be reasonable and rational	3.08 ± 4.8 (0.0)
Child should understand the concept of altruism	1.92 ± 3.3 (0.0)
Requirements for meaningful assent: older children (12-17 yrs)	
	*Ranking* *Mean ± SD (Median)*
Child should understand the basic information	22.23 ± 10.6 (20.0)
Child should be able to appreciate the information (i.e., apply the information to their own situation)	15.88 ± 4.5 (17.5)
Child should have a developmentally appropriate awareness of their condition	14.53 ± 5.6 (10.0)
Child should be able to articulate an unambiguous choice	13.84 ± 5.5 (10.0)
Decisions should be free of any external pressure or expectations	11.92 ± 8.3 (10.0)
Child should be able to reason (i.e., able to incorporate information with personal priorities and potential consequences)	9.62 ± 6.91 (10.0)
Decisions must be reasonable and rational	5.11 ± 6.0 (5.0)
Child should understand the concept of altruism	3.00 ± 4.6 (3.0)

Seventeen (94%) panelists agreed with the final definitions of capacity for assent, information disclosure for younger children, and the components of meaningful assent, respectively. Fifteen (83%) members agreed with the information disclosure for adolescents/older children. The final consensus versions of each of these constructs are shown below.

### Assessment of child capacity

*Assessment of the general development of the child can typically be accomplished through a discussion with either the child alone or together with a parent or legal guardian to gauge maturity/cognitive ability. Consideration should be given to the child’s current health status and any prior decision-making experiences. Assessment should employ a “teach back” method to assess developmentally appropriate understanding of the study information. In cases in which capacity is in doubt, it may be appropriate to involve a child-behavioral specialist.*

### Information for younger children (7-11 yrs)

*If assent is deemed appropriate, younger children should, at minimum, be told what procedures will be done and how the child might experience them, the purpose of the study, that there may be no expectation of personal benefit but that their participation may help other children, that the study is voluntary, and that they can withdraw at any time.*

### Information for older children/adolescents (12-17 yrs)

*At minimum, older children and adolescents should be told what procedures will be done and how they might experience them, the purpose of the study, that there may be no expectation of personal benefit but that their participation may help other children, that the study is voluntary, and that they can withdraw at any time. In some cases, it may appropriate for the investigator to speak with the child or adolescent absent from his or her parent(s) or legal guardian(s). For some therapeutic trials it is important that older children and adolescents be told that the research may be different from standard clinical care in their situation.*

### Requirements for meaningful assent (younger and older children definitions were collapsed)

*If assent is deemed appropriate, children should understand the basic study-specific information, should have a developmentally appropriate awareness of their condition, and be able to appreciate, at a rudimentary level, how the information applies to their own situation. Children should be free to decide whether or not to participate in a study and articulate their choice absent of any undue influence or coercion.*

## Discussion

Child assent serves as an important protection for children in research [24] however, the current regulations offer no single standardized operational definition of assent and no guidance regarding *which* children may be capable of providing it [21]. Importantly, there have also been no systematic efforts to define the constructs of assent. Indeed, Sibley et al. [25] wrote: “The definition, purpose or scope of assent (like the interpretation of dissent) remains woefully ill-defined.” To this end, we convened a panel of expert stakeholders who through iterative review reached consensus on several important definitions of assent for research. To our knowledge this is the first study to attempt to standardize the definition of assent using this approach. While we fully acknowledge that the information in our operational definition is not necessarily novel in that it can be sourced from a review of the extant literature, our aim was to provide a single concise statement that incorporated the important elements of the assent process not addressed in the current definitions.

While the most widely adopted definition of assent as a child’s “affirmative agreement” is fundamentally correct, it lacks details regarding the circumstances under which assent should be sought and details of what information should be conveyed and understood. This has likely led to the lack of consensus among stakeholders which as Sibley et al. suggest may in part be due to the fact that: “the fundamental justification for the assent process is misunderstood” [3]. While the traditional approach has been how to involve parents and children in the assent process, these authors argue that a primary goal should be to respect children by recognizing their moral worth and helping them become better decision-makers [3].

Delphi has several advantages over conventional qualitative methods i.e., inexpensive, avoids meeting logistics, decreases group think, the bandwagon effect, and disputes between personalities with widely disparate view-points [12]. Relevant to this study, Buccini et al. and Geisser et al. used Delphi to define the constructs of consent comprehension [16] and to establish the dimensions of ethics/IRB review, respectively [23]. For this study, we used the Delphi approach to build upon our original conceptualization and definitions of assent. Although the central theme of “affirmative agreement” was retained, each round of reviews added context with respect to the importance of assent as an “interactive” process, the circumstances in which assent should be sought, the need for age-appropriate disclosure, and the importance for children to understand their role and rights in a clinical trial.

The amount and type of information to be disclosed to children of different ages/maturity have not previously been well described. This study provided some insight into which elements of disclosure are deemed most important but also reinforced the importance of age-appropriate disclosure that takes into consideration the cognitive and emotional maturity of the child. As Hein et al. has shown, some children over the age of 12 years may be capable of providing their own consent for research but due to the inherent variability in maturity levels, the type and amount of information disclosed and the assessment of capacity should be considered on a case by case basis [10]. Of note, was that although we had originally included the “risks” of research participation as an important element of disclosure, the panel chose to rephrase this to reflect how the child might experience the risks rather than risks per se. We believe that this better reflects the way in which children often perceive risks i.e., as a burden: will it hurt and/or how might this affect me; will it keep me away from my usual activities and friends?

Although it may not be possible to cover all the nuances of assent or the many circumstances under which it may be sought within a single definition, we attempted to examine assent within the contexts of different age/maturity levels and potential risk profiles. Interestingly, it became quite evident that definitions of assent within these contexts had more common themes than disparate and, as such, could be collapsed into single definitions.

Limitations of this approach are recognized. First, our study represents the views from one group of experts interpreted by one group of investigators, and while it is likely that another similarly expert panel would arrive at similar conclusions, we cannot be certain. Second, because we believed it important to understand how the definitions might change with maturity level, we chose 11 years as a developmental cut-point. Although we justify this cut-point, it can only be considered a surrogate assessment of maturity and, as such, the results should be interpreted accordingly. Third, while Delphi offers several advantages, it does not allow for in-depth discussions. We should note however, that we were encouraged by the depth and detail of the many individual viewpoints. Furthermore, while thematic analysis highlighted the most salient points, items that did not fit a common theme may have been excluded in the definitions offered for review. Thus, while we made every attempt to include as many themes that we believed to be relevant, we acknowledge that it is almost impossible within any single definition to account for all the circumstances under which assent is sought and that aligns perfectly with everyone’s views and beliefs. Our final definitions were thus attempts to be inclusive yet concise. Another potential limitation was that while we had initially considered including children as stakeholders in the Delphi panel, we were concerned that children lacked the requisite ethical expertise and ability to meaningfully complete the Delphi questionnaire and assign relative percentage values to each of the elements. Finally, although we had sought input from experts outside of the US, unfortunately, none responded. While we believe that our definitions could be relevant outside of the US, additional studies may be warranted.

## Conclusions

While we acknowledge that not all definitions can be all things to all people, we hope that our proposed consensus-driven definitions will inform a more comprehensive and practical characterization of the assent process compared with current definitions and serve as a platform for further discussion and thought. Although this study is focused on assent for research, we also believe that the results have important implications for the way in which clinical assent is defined and operationalized. We hope therefore, that in providing a single operational definition of assent that incorporates elements deemed most important by stakeholders that we may be able to positively inform the way in which investigators, regulators, and IRBs operationalize the assent process, respect children’s developing autonomy and decision-making skills, and in concert with parental permission, ensure the protection of children participating in research.

## Additional files

Additional file 1: Iterative panel reviews of an operational definition of assent. (DOCX 18 kb)

Additional file 2: Iterative panel reviews of the construct definitions of assent for older and younger children in therapeutic and non-therapeutic trials. (DOC 26 kb)
