# Supplementary material for: Development of a consensus operational definition of child assent for research
Source: BMC Med Ethics. 2017 Jun 9;18:41. doi: 10.1186/s12910-017-0199-4 (PMC5466722; doi:10.1186/s12910-017-0199-4)
Supplement: Supplementary file 1 — Iterative panel reviews of an operational definition of assent. (DOCX 18 kb) [file 12910_2017_199_MOESM1_ESM.docx]

**Additional File 1:** Iterative panel reviews of an operational definition of assent

**1a. Operational definition – 1^st.^ revision**

*Children who lack the maturity or legal authority to provide informed consent must provide their assent to participate in a potential research study. Assent is an interactive process between a researcher and child participant involving disclosure of cognitively and emotionally appropriate information regarding, at minimum, what the child might expect, an understanding that participation in the study is voluntary, and knowledge that he or she can decline participation or withdraw at any time. Valid assent requires that the child explicitly affirms his or her agreement to participate free of any outside influences, pressure, or coercion. In the absence of an explicit agreement, mere failure of the child to object cannot be construed as assent.*

**1b. Operational definition – 2^nd.^ revision**

*Children who lack the legal authority to provide informed consent must provide their assent to participate in a research study unless they either lack the cognitive ability or their clinical condition precludes meaningful participation. Assent is an interactive process between a researcher and child participant involving disclosure of cognitively and emotionally appropriate information regarding, at minimum, why the child is being asked to participate, what the child might expect from the study, an understanding that participation in the study is voluntary, and knowledge that he or she can decline participation or withdraw at any time. Valid assent requires that the child explicitly affirms his or her agreement to participate in a manner that reflects age appropriate understanding, is reasonable, rational, and free of any coercion. In the absence of an explicit agreement, mere failure of the child to object cannot be construed as assent.*

**1c. Operational definition – 3^rd.^ revision**

*Children who lack the legal authority to provide informed consent (i.e., are not emancipated under state law or are under 18 years of age) should provide their assent to participate in a research study unless they either lack the cognitive ability, their clinical condition precludes their ability to communicate a choice, or the research holds out the prospect of direct benefit that is only available in the context of the research. Assent is an interactive process between a researcher and child participant involving disclosure of cognitively and emotionally appropriate information regarding, at minimum, why the child is being asked to participate, a description of the procedures and how the child might experience them, and an understanding that participation in the study is voluntary. Children should understand that they can decline participation or withdraw from the study at any time but in doing so should be aware of any potential consequences of non-participation. Valid assent requires that the child explicitly affirms his or her agreement to participate in a manner that reflects age appropriate understanding and that is free of undue influence or coercion. In the absence of an explicit agreement, mere failure of the child to object cannot be construed as assent.*
