# Supplementary material for: Development of a consensus operational definition of child assent for research
Source: BMC Med Ethics. 2017 Jun 9;18:41. doi: 10.1186/s12910-017-0199-4 (PMC5466722; doi:10.1186/s12910-017-0199-4)
Supplement: Supplementary file 2 — Iterative panel reviews of the construct definitions of assent for older and younger children in therapeutic and non-therapeutic trials. (DOC 26 kb) [file 12910_2017_199_MOESM2_ESM.doc]

**Additional file 2:** Iterative panel reviews of the construct definitions of assent for older and younger children in therapeutic and non-therapeutic trials

**2a. Construct definitions – Preliminary definitions based on summary rankings**

**Assessment of child capacity**

*Assessment of the general development of the child can be accomplished through a discussion with both the parent(s) and the child to gauge maturity/cognitive ability. Assessment should employ a “teach back” method to assess developmentally appropriate understanding of the study information.*

**Information for younger children (7-11 yrs)**

*If assent is deemed appropriate, younger children should, at minimum, be told what will be done (the procedures), the purpose of the study, that the study is voluntary, that they can withdraw at any time, and any potential benefit to themselves.*

**Information for older children (12-17 yrs)**

*At minimum, older children and adolescents should be told what will be done (the procedures), the purpose of the study, that the study is voluntary, that they can withdraw at any time, the risks of the study, and the potential benefits to themselves and other children. For some therapeutic trials it is important that older children and adolescents understand that research may be different from clinical care.*

**Requirements for meaningful assent (younger and older definitions were collapsed)**

*If assent is deemed appropriate, children should be able to understand the basic study-specific information, should have a developmentally appropriate awareness of their condition, and be able to appreciate the information (i.e., apply the information to their own situation). Children should be free to make decisions absent of any coercion and be able to articulate an unambiguous choice. Older children and adolescents may also be required to demonstrate the ability to reason (i.e., incorporate information with personal priorities and potential consequences).*

**2b. Construct definitions – 1^st^. revision**

**Assessment of child capacity**

*Assessment of the general development of the child can typically be accomplished through a discussion with either the child alone or together with a parent to gauge maturity/cognitive ability. Consideration should be given to the child’s current health status and any prior decision-making experiences. Assessment should employ a “teach back” method to assess developmentally appropriate understanding of the study information. In cases in which capacity is in doubt, it may be appropriate to involve a child-behavioral specialist and/or standardized testing.*

**Information for younger children (7-11 yrs)**

*If assent is deemed appropriate, younger children should, at minimum, be told what procedures will be done and how the child might experience them, the purpose of the study, that there may be no expectation of personal benefit but that their participation may help other children, that the study is voluntary, and that they can withdraw at any time with an understanding of the potential consequences (if any) of non-participation.*

**Information for older children (12-17 yrs)**

*At minimum, older children and adolescents should be told what procedures will be done and how they might experience them, the purpose of the study, that there may be no expectation of personal benefit but that their participation may help other children, that the study is voluntary, and that they can withdraw at any time with an understanding of the potential consequences (if any) of non-participation. In some cases it may appropriate for the investigator to speak with the child or adolescent absent from his or her parent(s). For some therapeutic trials it is important that older children and adolescents understand how the research may be different from standard clinical care in their situation.*

**Requirements for meaningful assent (younger and older definitions were collapsed)**

*If assent is deemed appropriate, children should be able to understand the basic study-specific information, should have a developmentally appropriate awareness of their condition, and be able to appreciate, at a rudimentary level, how the information applies to their own situation. Children should be free to decide whether or not to participate in a study and articulate their choice absent of any undue influence or coercion.*
